# The ambulatory arterial stiffness index and target-organ damage in Chinese patients with chronic kidney disease

**DOI:** 10.1186/1471-2369-14-257

**Published:** 2013-11-19

**Authors:** Cheng Wang, Jun Zhang, Cui-Cui Li, Wen-Yu Gong, Xun Liu, Zeng-Chun Ye, Hui Peng, Tan-Qi Lou

**Affiliations:** 1 Division of Nephrology, Department of medicine, Third Affiliated Hospital of Sun Yat-Sen University, Guangzhou, Guangdong 510630, China

**Keywords:** Ambulatory arterial stiffness index, Chronic kidney disease, Renal function, Left ventricular mass index

## Abstract

**Background:**

The ambulatory arterial stiffness index (AASI) can be used to predict cardiovascular morbidity and mortality in hypertensive patients. However, data on AASI in Chinese patients with chronic kidney disease (CKD) is not available.

**Methods:**

This cross-sectional study enrolled 583 CKD patients. Univariate and multivariate analyses were used to evaluate the relationship between AASI and renal function and parameters of cardiovascular injury.

**Results:**

Patients with a higher AASI had a higher systolic blood pressure, a lower estimated glomerular filtration rate (eGFR), a higher serum cystatin C, a higher left ventricular mass index (LVMI) and carotid intima-media thickness (cIMT). Univariate analyses showed that AASI was positively correlated with serum cystatin C (*r*=0.296, *P* < 0.001), serum creatinine (*r*=0.182, *P* < 0.001), and LVMI (*r* = 0.205, *P* < 0.001) and negatively correlated with the eGFR (*r* = –0.200, *P* < 0.001). Multivariate analyses revealed that serum cystatin C, eGFR, serum creatinine and LVMI were independently correlated with AASI.

**Conclusions:**

These data suggest that AASI was closely correlated with renal function and parameters of cardiovascular injury in Chinese CKD patients. Good quality, long-term, large longitudinal trials to validate the role of AASI in clinical practice for Chinese CKD patients.

## Background

The rising prevalence and associated morbidity of chronic kidney disease (CKD) has resulted in a significant disease burden and became a major public health problem for many countries [1]. Cardiovascular disease (CVD) is the leading cause of premature death in patients with CKD [2], so research on vascular changes in CKD patients is very important. Reduced arterial elasticity has been observed in CKD patients [3]. Fibroelastic intimal thickening, an increased extracellular matrix, enhanced collagen density, and vascular calcification seem to contribute to “stiffer” arteries [4]. It has been demonstrated that vascular stiffness can predict adverse cardiovascular outcomes in patients with primary hypertension [5].

In 1914, MacWilliam and Melvin stated that a loss of elasticity in the arterial system influences diastolic blood pressure (DBP) and its relationship with systolic blood pressure (SBP). According to this basic principle, Li et al. proposed a novel, easy-to-obtain index of arterial stiffness: the ambulatory arterial stiffness index (AASI). AASI is defined as 1 minus the regression slope of DBP plotted against SBP obtained from individual 24-h blood pressure (BP) recordings. They also described the close correlation of AASI with pulse wave velocity (PWV) as well as central and peripheral augmentation indices [6]. Moreover, Dolan et al. showed that AASI can provide prognostic information because it was a predictor of stroke and cardiac death in a cohort of 11,291 patients [7]. Kikuya et al. also observed that the AASI predicted mortality due to CVD and stroke over and beyond pulse pressure [8]. Muxfeldt et al. demonstrated that AASI was a predictor of cardiovascular morbidity and mortality in 547 patients with resistant hypertension [9]. Furthermore, some research teams have reported that AASI has good reproducibility, with repeatability coefficients of ≤60% [10]. AASI has good correlation with target-organ damage in patients with primary hypertension [11,12]. However, some have criticized the significance of AASI in assessing arterial compliance, especially in pediatric populations [13,14]; what is more, few studies have focused on AASI in Chinese CKD patients. Investigating the relationship between AASI and target organ damage in Chinese CKD patients is very important when considering different life style, genetic factors, environment and the primary cause of CKD in the Chinese population. Hence, we carried out an observational study to identify the relationship between AASI and target organ damage in CKD patients residing in China.

## Methods

### Design and population of the study

The study protocol was approved by the ethics committee of 3^rd^ Hospital of Sun Yat-sen University. All of the study participants provided written informed consent to be included in the study. Six hundred and fifty consecutive in patients with CKD were included from May 2010 to July 2012. A cross-sectional study was carried out in our division. The exclusion criteria were: treatment with corticosteroids or hormones; acute changes in the estimated glomerular filtration rate (eGFR) >30% in the previous 3 months; pregnancy; history of abuse of drugs or alcohol; night or shift-work employment; acquired immunodeficiency syndrome; cardiovascular disorders (unstable angina pectoris, heart failure, life-threatening arrhythmia, atrial fibrillation and grade III–IV retinopathy); intolerance to ambulatory blood pressure monitoring (ABPM); inability to communicate and comply with all of the study requirements; or maintenance dialysis. We excluded 67 patients for abnormal ABPM or incomplete measurement of baseline characteristics. Therefore, 583 patients were enrolled into the study. Forty-nine subjects had diabetic nephropathy and 109 patients had immunoglobulin (Ig) A nephropathy in this group.

### Assessment

1. ABPM: Patients underwent 24-h ABPM using a TM-2430 monitor (A&D, Tokyo, Japan). Cuff size was chosen based on arm circumference. The cuff was fixed to the non-dominant arm. Three BP readings were obtained in the morning (07:00 to 10:00) concomitant with sphygmomanometric measurements to ensure that the average of the two sets of values differed by ≤5 mmHg. BP was recorded every 15 min from 07:00 to 22:00, and from every 30 min from 22:00 to 07:00. The values for daytime and nighttime periods were derived from diaries recorded by the patients during ABPM. Monitoring was done on a working day. Patients had no access to ABP values. Strenuous physical activity was discouraged in all patients during the monitoring period, and their daily activities were comparable.

BP series were eliminated from the analysis if: >30% of the measurements were lacking; they had missing data for >3-h spans; they were collected from subjects who were experiencing an irregular rest–activity schedule or a nighttime sleep span <6 h or >12 h during monitoring. Raw data were examined by scatter plots. The regression slope of DBP on SBP was computed to obtain the AASI (1-slope). The diagnosis of hypertension was based on accepted criteria for ABPM [15]: ambulatory blood pressure was considered to be “normal” if the mean awake BP was <135/85 mmHg for SBP/DBP or if the mean bedtime BP was <120/70 mmHg.

2. BP measurement in the physician’s office: BP was measured during a visit to the physician’s office (8 am to 11 am) [16]. Briefly, measurements were made in a quiet environment using a mercury sphygmomanometer with the patient in a sitting position after 5 min of rest. BP was not measured if the patient had engaged in recent physical activity, used tobacco, ingested caffeine, or eaten within the previous 30 min. Values of SBP and DBP values (Korotkoff’s phase I and phase V, respectively) in each visit were used to obtain a minimum of two BP measurements at intervals of ≥1 min. For each visit (baseline and after the drug shift), the reported values of office BP were the mean of values recorded during the 2 days in which the ABP device was installed and removed. For all patients, sphygmomanometric measurements were undertaken by the same physician, who was not aware of the results of ABP recordings.

3. Cardiac assessment: Cardiac structure was assessed by two investigators trained for this purpose before starting the study. Left ventricular volumes, mass, systolic function and diastolic function were assessed using 2-dimensional echocardiography. Left ventricular mass was calculated using the Duvereux method [17]. The left ventricular mass index (LVMI) was obtained by calculating left ventricular mass to height^2.7^[18]. Left ventricular systolic function was assessed by left ventricular ejection fraction, and diastolic function was assessed by early mitral inflow filling velocity (E), peak mitral filling velocity at atrial contraction (A), E/A deceleration time of the mitral E wave and tissue Doppler velocity of the mitral annulus [19].

4. Carotid ultrasonography: Carotid intima-media thickness (cIMT) was assessed by two trained investigators before study commencement. A SonoSite MicroMaxx Ultrasound System paired with a 5–10-MHz Multifrequency High-resolution linear transducer (Bothell, WA, USA) with Sono-Calc IMT software was used for taking automatic measurements of cIMT. This was achieved by averaging three measurements taken on each carotid artery (anterior, lateral and posterior directions) and measuring the distance between the leading edge of the lumen–intima interface and the leading edge of the collagenous upper layer of the adventitia using high-resolution B-mode ultrasonography. Measurements were taken in areas free of obvious atherosclerotic plaque around the level of the carotid bifurcation.

5. Renal assessment: Kidney damage was assessed by measuring the serum concentrations of creatinine, which is measured by the enzymatic method, traceable to the isotope dilution mass spectrometry. The estimated glomerular filtration rate (eGFR) was calculated using a modified version of the Modification of Diet in Renal Disease (MDRD) equation based on data from Chinese CKD patients as follows [20]:

$$\mathrm{eGFR}=175\times \mathrm{standardized}\;\mathrm{Sc}r^{‒1.234}\times \mathrm{ag}e^{-0.179}\times 0.79\left(\mathrm{if}\;\mathrm{female}\right)$$

6. Other data collection: We also collected urine samples from 07:00 to *0*7:00 the next day to detect the extent of 24-h proteinuria. These patients were asked to void their bladders at *0*7:00 to ensure valid results. Proteinuria was measured by immunoturbidimetry. In addition, medical history, including demographic and laboratory data [serum cystatin C, cholesterol, triglycerides (TGs), high-density lipo-protein-cholesterol (HDL-C), low-density lipoprotein-cholesterol (LDL-C), fasting glucose, homocysteine, calcium , phosphate, and intact parathyroid hormone (iPTH)] as well as current therapy were obtained at the initial study visit. The experimental data were measured using a Hitachi 7180 biochemistry auto-analyzer (Tokyo, Japan).

### Statistical analyses

Continuous variables were expressed as the mean ± standard deviation (SD). Frequency distributions were used for qualitative variables. Non-parametric variables are expressed as median and interquartile range. The log transformation for proteinuria was done in view of the skewed distribution of these data, and Ln (proteinuria + 1) was used because some patients had low levels of proteinuria.

One-way analysis (ANOVA) or non-parametric test was used to analyze differences among the quartiles of AASI distribution. Pearson’s correlation coefficient was employed to estimate the relationship between quantitative variables, and the chi-squared test was used to associate qualitative variables. We employed multivariate linear regression models to study the association of indices of renal function (levels of cystatin C, serum creatinine, eGFR and proteinuria) and cardiovascular damage (LVMI, E/A ratio, cIMT) with AASI values. All values are two-tailed. *p* < 0.05 was considered significant. Data were analyzed using SPSS ver18.0 (SPSS, Chicago, IL, USA).

## Results

1 Clinical characteristics of patients

Of 583 CKD patients (mean age, 43 ± 16 years; male/female ratio, 1.45) only 49 (8.4%) had diabetes mellitus (DM) but 109 (18.7%) had IgA nephropathy. The mean AASI was 0.54 ± 0.17. Univariate analyses showed that AASI was positively related with age (*r* = 0.263, *P* < 0.001). The AASI in patients with IgA nephropathy was 0.47 ± 0.18, which was lower than non-IgA nephropathy patients (*P* < 0.05). The AASI in DM patients was 0.57 ± 0.11, which was similar to that seen in non-DM patients.

The main clinical characteristics of the study population across the quartiles of AASI distribution are shown in Table 1. Patients with a higher AASI were older, and had: higher SBP (in the clinic, over 24-h, at daytime and at bedtime); higher mean BP; smaller variability in DBP (mean, at daytime and at bedtime); lower eGFR; higher serum levels of cystatin C; lower levels of hemoglobin; higher serum levels of phosphate; iPTH and calcium × phosphate; higher LVMI and cIMT and a higher prevalence of carotid arterial plaques.

2 AASI is closely correlated with clinical parameters

Univariate analyses showed that AASI was positively correlated with: serum levels of phosphate (*r* = 0.178, *P* < 0.001); iPTH (*r* = 0.179, *P* < 0.001) and calcium × phosphate (*r* = 0.163, *P* < 0.001); serum levels of uric acid (*r* = 0.107, *P* = 0.01), and negatively correlated with levels of hemoglobin (*r* = –0.203, *P* < 0.001) and HDL-C (*r* = –0.096, *P* = 0.025) (Table 2).

Univariate analyses showed that AASI was positively correlated with SBP in the clinic (*r* = 0.168, *P* < 0.001), over 24 h (*r* = 0.311, *P* < 0.001), while at daytime (*r* = 0.322, *P* < 0.001) and bedtime (*r* = 0.196, *P* < 0.001). It was also positively correlated with mean BP (*r* = 0.226, *P* < 0.001) and heart rate (*r* = 0.09, *P* = 0.017). AASI was negatively correlated with the variability of DBP over 24 h (*r* = –0.188, *P* < 0.001), while at daytime(*r* = –0.283, *P* < 0.001) and bedtime (*r* = –0.162, *P* < 0.001). AASI was also negatively correlated with SBP while at daytime (*r* = –0.114, *P* = 0.006) and bedtime (*r* = –0.091, *P* = 0.029) as well as the decline in bedtime SBP (*r* = –0.124, *P* = 0.003) and DBP (*r* = –0.226, *P* < 0.001) (Table 2).

3 AASI is closely correlated with renal function

Univariate analyses revealed AASI was negatively correlated with the eGFR (*r* = –0.200, *P* < 0.001) and positively correlated with serum levels of cystatin C (*r* = 0.296, *P* < 0.001) and serum creatinine(*r* = 0.182, *P* < 0.001) Multiple linear regression analyses were done to evaluate the relationship between eGFR, serum levels of cystatin C and creatinine with AASI. The eGFR was independently correlated with age (β = -1.477), 24-h mean BP (β = -1.538), the decline ratio of bedtime SBP (β =0.534) and the AASI (β = –20.033). Serum levels of cystatin C were independently correlated with age (β = 0.022), 24-h mean BP (β =0.045), the decline in the ratio of bedtime SBP (β = -0.038), and AASI (β = 1.795). Serum creatinine were independently correlated with sex (β = -91.657), 24-h mean BP (β = 9.259), the decline in the ratio of bedtime SBP (β = -8.568) and AASI (β = 262.347). A significant relationship between AASI and proteinuria was not found when univariate and multiple linear regression analyses were undertaken (Tables 2 and 3).

4 AASI is closely correlated with the LVMI

Univariate analyses showed that AASI was positively correlated with the LVMI (*r* = 0.205, *P* < 0.001) and cIMT (*r* = 0.156, *P* = 0.002), and was negatively related to the E/A ratio (*r* = –0.114, *P* = 0.018). However, we found that the LVMI (but not the cIMT and E/A ratio) was independently correlated with AASI (β = 1.712) after multiple linear regression analyses were carried out (Table 4).

**Table 1:** Characteristics of the study population by quartiles of AASI distribution

**Variables**	**Quartile1**	**Quartile2**	**Quartile3**	**Quartile4**
	**(<0.42)**	**(0.42 ~ 0.53)**	**(0.54 ~ 0.63)**	**(>0.63)**
	**(n = 145)**	**(n = 146)**	**(n = 146)**	**(n = 146)**
Age (years)	36 ± 14	43 ± 16*	44 ± 17*#	48 ± 17*#&
Male:female ratio	81: 59	87:59	87:60	90:60
Number of diabetic	6	11	15	17
ACEIs or ARBs (%)	50.4	55.2	58.1	63.3
CCB (%)	18.4	21.5	24.6	27.8
Proteinuria (g/24h)	1.2(0.4-4.1)	1.3(0.5-3.3)	1.4(0.5-3.9)	1.2(0.6-3.2)
Hemoglobin (g/L)	121 ± 29	114 ± 30	113 ± 30*	105 ± 31*#&
Albumin (g/L)	33 ± 9	35 ± 22	33 ± 9	35 ± 8
Globulin (g/L)	23 ± 5	24 ± 5	24 ± 6	25 ± 6
Serum creatinine (μmol/L)	235 ± 215	329 ± 216*	338 ± 261*#	445 ± 254*#&
Serum Cystatin C (mg/L)	1.9 ± 1.5	2.4 ± 1.8*	2.6 ± 2.0*#	3.5 ± 2.5*#&
eGFR-MDRD (ml/min/1.73m^2^)	81(22-115)	51(10-103)*	47(12-102)*	27(6-89)*#&
Cholesterol (mmol/L)	6.3 ± 3.1	6.2 ± 3.0	6.2 ± 3.5	5.5 ± 2.8
Triglyceride (mmol/L)	2.1 ± 1.5	2.2 ± 1.4	2.5 ± 2.4	2.0 ± 1.9
LDL-C (mmol/L)	4.1 ± 2.5	4.0 ± 2.0	3.9 ± 2.5	3.5 ± 2.1
HDL-C (mmol/L)	1.3 ± 0.5	1.2 ± 0.5	1.2 ± 0.5	1.2 ± 0.4
Glucose (mmol/L)	5.0 ± 1.8	5.1 ± 1.9	5.2 ± 1.8	5.3 ± 2.0
Calcium (mg/dL)	8.6 ± 0.8	8.7 ± 0.9	8.4 ± 1.0#	8.5 ± 0.9
Phosphate (mmol/L)	4.1 ± 1.1	4.5 ± 1.6*	4.5 ± 1.6*	4.9 ± 1.8*#&
Calcium × Phosphate (mg^2^/dl^2^)	35 ± 8	39 ± 13*	37 ± 10	41 ± 13*&
iPTH (pg/ml)	116 ± 98	139 ± 94	155 ± 130*	217 ± 167*#&
Urine acid (mmol/L)	433 ± 150	460 ± 156	466 ± 146	470 ± 157
homocysteine (μmol/L)	14.3 ± 8.6	18.2 ± 10.0*	16.8 ± 8.4*	16.2 ± 7.9*
LVEF (%)	63 ± 18	64 ± 16	66 ± 12	65 ± 14
LVMI (g/m^2.7^)	43 ± 15	49 ± 17*	56 ± 27*	54 ± 19*
E/A ratio	1.3 ± 0.4	1.1 ± 0.4*	1.2 ± 0.6	1.1 ± 0.4*
cIMI (mm)	0.61 ± 0.22	0.64 ± 0.21	0.68 ± 0.25	0.73 ± 0.35*#
Number of plaque	6	10	19*	20*
Clinic- SBP (mmHg)	135 ± 21	141 ± 23*	146 ± 24*	145 ± 26*
Clinic-DBP (mmHg)	84 ± 14	85 ± 15	87 ± 14	84 ± 14
average-SBP (mmHg)	126 ± 17	131 ± 20*	138 ± 20*#	142 ± 20*#
average-DBP (mmHg)	78 ± 11	79 ± 12	82 ± 12	81 ± 11
SBP-daytime (mmHg)	128 ± 17	134 ± 18*	138 ± 19*#	143 ± 19*#&
DBP-daytime (mmHg)	79 ± 11	81 ± 11	82 ± 12	82 ± 11
SBP-bedtime (mmHg)	110 ± 31	122 ± 39*	128 ± 31*	135 ± 28*#&
DBP-bedtime (mmHg)	67 ± 19	74 ± 16*	77 ± 20*	78 ± 15*#
Decline of SBP-bedtime	7.9 ± 7.0	6.3 ± 6.0	6.0 ± 14	4.5 ± 11.4
Decline of DBP-bedtime	10.6 ± 8.5*	7.2 ± 8.9*	6.6 ± 13.6*	3.6 ± 11.5*&
24h-MBP	93 ± 12	96 ± 14	100 ± 13	101 ± 113*#
Variability of SBP (%)	14 ± 8	15 ± 8	15 ± 11	13 ± 8
Variability of DBP (%)	24 ± 20	20 ± 9*	18 ± 10*	16 ± 6*#
Variability of SBP-daytime (%)	13 ± 5	14 ± 5	13 ± 4#	12 ± 4#
Variability of DBP -daytime (%)	21 ± 7	19 ± 7*	17 ± 5*#	16 ± 5*#&
Variability of SBP-bedtime (%)	12 ± 6	12 ± 5	11 ± 6	10 ± 5*#
Variability of DBP -bedtime (%)	16 ± 7	16 ± 7	14 ± 6*#	13 ± 5*#
Pulse (beat/min)	75 ± 10	78 ± 10*	78 ± 11	78 ± 11
AASI (IU)	0.32 ± 0.08	0.48 ± 0.03*	0.59 ± 0.03*#	0.74 ± 0.08*#&

(AASI: ambulatory arterial stiffness index, ARB: angiotensin receptor blocker, ACEI: angiotensin-converting enzyme inhibitors, BMI: body mass index, CCB: calcium channel blocker, eGFR: estimated glomerular filtration rate; LDL-C: low-density lipoprotein cholesterol; HDL-C: high-density lipoprotein cholesterol; iPTH: intact parathyroid hormone; LVEF: Left ventricular ejection fraction; LVMI: Left ventricular mass index; E: early mitral inflow filling velocity; A: peak mitral filling velocity at atrial contraction; cIMI: Carotid intima-media thickness. Serum calcium is corrected by the following formula: [calcium] (mg/dL) = measured [calcium] + ( 4.0-[serum albumin (mg/dl)]) × 0.8; SBP: systolic blood pressure; DBP: diastolic blood pressure; ABPM: ambulatory blood pressure monitoring. *indicated contro with Quartile1 P < 0.05, # indicated contro with Quartile2 P < 0.05, &# indicated contro with Quartile3 P < 0.05).

**Table 2:** Baseline characteristics of clinical data and correlation coefficient with AASI

**Variables**	**N = 583**	**Correlation coefficient with AASI**	**P value**
Age (years)	43 ± 16	0.263	<0.001
Duration (months)	24 ± 17	0.075	0.074
BMI (kg/m^2^)	23 ± 4	−0.019	0.652
Ln (Proteinuria + 1)	1.0 ± 0.7	−0.007	0.867
Hemoglobin (g/L)	113 ± 30	−0.203	<0.001
Albumin (g/L)	34 ± 13	0.004	0.931
Globulin (g/L)	24 ± 6	0.098	0.02
Serum Cystatin C (mg/L)	2.6 ± 2.1	0.296	<0.001
Serum creatinine (μmol/L)	339 ± 222	0.182	<0.001
eGFR-MDRD (ml/min/1.73m^2^)	59 ± 48	−0.200	<0.001
Cholesterol (mmol/L)	6.1 ± 3.1	−0.089	0.039
Triglyceride (mmol/L)	2.2 ± 1.8	−0.028	0.517
LDL-C (mmol/L)	3.9 ± 2.4	−0.08	0.062
HDL-C (mmol/L)	1.2 ± 0.5	−0.096	0.025
Glucose (mmol/L)	5.2 ± 1.5	0.079	0.057
Calcium (mg/dL)	8.6 ± 0.8	−0.079	0.056
Phosphate (mmol/L)	4.5 ± 1.6	0.178	<0.001
Calcium × Phosphate (mg^2^/dl^2^)	36 ± 10	0.163	<0.001
iPTH (pg/ml)	157 ± 73	0.179	<0.001
Uric acid (mmol/L)	458 ± 163	0.107	0.010
homocysteine (μmol/L)	16.4 ± 8.8	0.067	0.143
LVEF (%)	63 ± 16	0.036	0.445
LVMI (g/m^2.7^)	51 ± 21	0.205	<0.001
E/A ratio	1.2 ± 0.5	−0.114	0.018
cIMI (mm)	0.7 ± 0.3	0.156	0.002
Clinic-SBP (mmHg)	142 ± 24	0.168	<0.001
Clinic-DBP (mmHg)	85 ± 14	−0.006	0.881
average-SBP (mmHg)	134 ± 20	0.311	<0.001
average-DBP (mmHg)	80 ± 12	0.128	0.002
SBP-daytime (mmHg)	136 ± 19	0.322	<0.001
DBP-daytime (mmHg)	81 ± 11	0.166	0.005
SBP-bedtime (mmHg)	124 ± 31	0.296	<0.001
DBP-bedtime (mmHg)	74 ± 18	0.216	<0.001
Decline of SBP-bedtime	6 ± 11	−0.124	0.003
Decline of DBP-bedtime	7 ± 11	−0.226	<0.001
24h-MBP	98 ± 13	0.226	<0.001
Variability of SBP (%)	14 ± 9	−0.039	0.352
Variability of DBP (%)	19 ± 13	−0.188	<0.001
Variability of SBP-daytime (%)	13 ± 4	−0.114	0.006
Variability of DBP-daytime (%)	18 ± 6	−0.283	<0.001
Variability of SBP-bedtime (%)	11 ± 6	−0.091	0.029
Variability of DBP-bedtime (%)	15 ± 6	−0.162	<0.001
Pulse (beat/min)	77 ± 10	0.09	0.017
AASI (IU)	0.54 ± 0.17	1.000	<0.001

(AASI: ambulatory arterial stiffness index, BMI: body mass index, eGFR: estimated glomerular filtration rate; LDL-C: low-density lipoprotein cholesterol; HDL-C: high-density lipoprotein cholesterol; iPTH: intact parathyroid hormone; LVEF: Left ventricular ejection fraction; LVMI: Left ventricular mass index; E: early mitral inflow filling velocity; A: peak mitral filling velocity at atrial contraction; cIMI: Carotid intima-media thickness. Serum calcium is corrected by the following formula: [calcium] (mg/dL) = measured [calcium] + ( 4.0-[serum albumin (mg/dl)]) × 0.8; SBP: systolic blood pressure; DBP: diastolic blood pressure; ABPM: ambulatory blood pressure monitoring).

**Table 3:** Multiple linear regression analysis: relationship between renal parameters (eGFR, Serum Cystatin C, serum creatinine and proteinuira) and AASI in CKD patients

**Variables**	**Not standardized b**	**Confidence interval 95%**	**P value**
Dependent variable: eGFR by MDRD formula (Adjusted R^2^ = 0.322)			
Age (year)	−1.477	−1.685 ~ -1.263	<0.001
24h-MBP	−1.538	−1.770 ~ -1.291	<0.001
decline ratio of bedtime SBP	0.534	0.225 ~ -0.834	0.001
AASI (IU)	−20.033	−40.263 ~ -0.807	0.037
Dependent variable: Serum Cystatin C (Adjusted R^2^ = 0.304)			
Age (year)	0.022	0.013 ~ 0.032	<0.001
24h-MBP	0.045	0.034 ~ 0.057	<0.001
Decline ratio of bedtime SBP	−0.038	−0.054 ~ -0.021	<0.001
AASI (IU)	1.795	0.883 ~ 2.707	<0.001
Dependent variable: Serum creatinine (Adjusted R^2^ = 0.213)			
Sex	−91.657	−153.032 ~ -30.283	0.003
24h-MBP	9.259	6.940 ~ 11.579	<0.001
Decline ratio of bedtime SBP	−8.568	−11.744 ~ -5.392	<0.001
AASI (IU)	262.347	78.405 ~ 446.289	0.005
Dependent variable: ln(proteinuria + 1) (Adjusted R^2^ = 0.034)			
Age	−0.008	−0.012 ~ -0.004	<0.001
24h-MBP	0.009	0.004 ~ 0.014	<0.001

Adjusted Variables: Age; Sex: (male = 1; female = 2).

Independent variable: ln (24Pro + 1), 24h-MBP, decline ratio of bedtime SBP, decline ratio of bedtime DBP, AASI (IU).

(AASI: ambulatory arterial stiffness index, eGFR: estimated glomerular filtration rate, MBP: mean blood pressure; SBP: systolic blood pressure).

**Table 4:** Multiple linear regression analysis: relationship between cardiovascular damage parameter (Left ventricular mass index, E/A ratio, Carotid intima-media thickness and Carotid aterial plaque) and AASI in CKD patients

**Variables**	**Not standardized β**	**Confidence interval 95%**	**P value**
Dependent variable: Left ventricular mass index (Adjusted R^2^ = 0.329)			
eGFR-MDRD (ml/min/1.73m^2^)	−0.143	−0.183 ~ -0.103	<0.001
BMI	1.392	1.020 ~ 1.764	<0.001
24h-MBP	0.163	0.012 ~ 0.313	0.034
decline ratio of bedtime SBP	−0.274	−0.451 ~ -0.096	0.003
AASI (IU)	1.712	0.191 ~ 3.233	0.027
Dependent variable: E/A ratio (Adjusted R^2^ = 0.283)			
Age	−0.014	−0.016 ~ -0.011	<0.001
24h-MBP	−0.006	−0.009 ~ -0.003	<0.001
Dependent variable: Carotid intima-media thickness (Adjusted R^2^ = 0.305)			
Age	0.009	0.008 ~ 0.010	<0.001
Sex	−0.070	−0.115 ~ -0.024	0.003

Adjusted Variables: Age; Sex: (male = 1; female = 2).

Independent variable: eGFR-MDRD (ml/min/1.73m^2^), BMI, 24h-MBP, decline ratio of bedtime SBP, decline ratio of bedtime DBP, AASI (IU).

(AASI: ambulatory arterial stiffness index, eGFR: estimated glomerular filtration rate, MBP: mean blood pressure; SBP: systolic blood pressure).

## Discussion

In this clinical trial, we initially explored the relationship between AASI and target-organ damage in CKD patients in China. The mean AASI in our patients was 0.54, which was higher than hypertensive patients in China [21]. Patients with a higher AASI were older and had: worse renal function; more chronic CKD (anemia, higher serum levels of phosphate and parathyroid hormone); higher LVMI and cIMT; and higher BP. AASI was correlated with renal function (eGFR and serum levels of cystatin C) and LVMI according to univariate and multivariate analyses. This finding suggested that the AASI was a potential index for the assessment of cardiovascular and renal damage. Furthermore, AASI was significantly correlated with variability in BP pressure, which suggested that AASI might be a parameter for assessing BP variability.

AASI is derived from BP parameters and thus the multiple reports on the correlation between AASI and pulse pressure or systolic BP do not necessarily reflect a true biological relationship. In a recent study using a computer model of the arterial circulation, arterial stiffness along with vascular resistance and heart rate were identified as the main determinants of AASI [22]. Moreover, several studies have shown night BP reduction to have considerable impact on AASI values [23]. Thus, AASI cannot be considered as a marker of arterial stiffness but rather as a composite index reflecting cardiovascular properties, BP variability and diurnal cycle. Our results showed AASI was correlated with target organ damage and BP variability in CKD patients. This is the first report on the relationship between AASI and target-organ damage in a large cohort of Chinese CKD patients based on cross-sectional data.

IgA nephropathy is the most common cause of primary glomerulonephritis in “developing” countries such as China [24], whereas diabetic nephropathy is the main cause of end stage of renal disease in developed countries [25]. Almost 20% patients in our study had IgA nephropathy, and less than 10% patients suffered from diabetic nephropathy, which is completely different from that seen in western countries. Patients with IgA nephropathy are younger, and show less vascular complications, whereas patients with diabetic nephropathy are older and have more micro and macro vascular complications. All these factors mentioned in Chinese CKD patients might lead to different roles of AASI in patients from western countries. We found that AASI in IgA patients was lower than non-IgA nephropathy patients, which was related to being younger and having slight renal damage. However the number of subjects with diabetic nephropathy in the present study was small, we need to study more diabetic patients to confirm the role of AASI in patients with diabetic nephropathy.

CVD is a leading cause of morbidity and mortality, accounting for 30-40% of deaths in patients with renal failure. Left ventricular hypertrophy, left ventricular dilatation, systolic dysfunction and diastolic dysfunction are very common and independently associated with mortality in CKD patients [26]. The cIMT is a marker for the presence and severity of arteriosclerosis, and has been associated with risk factors for all CVDs, all-cause mortality, and cardiovascular mortality [27]. AASI is a well-known predictor of cardiovascular mortality in hypertensive patients [28]. However, there are few reports on the relationship between AASI and cardiovascular damages. We are the first to study this relationship in Chinese CKD patients. We found that patients with higher AASI values had a higher LVMI and cIMT as well as a higher prevalence of carotid arterial plaques. Also, AASI was positively related to the LVMI and cIMT. Our results indicated that AASI was closely related to the traditional risk factors of CVD such as LVMI and cIMT. AASI might be a potential marker for the assessment of cardiovascular damage in CKD patients and more clinical trials are needed to confirm this finding.

Proteinuria is a strong, independent predictor of ESRD in a mass-screening setting. Even a slight increase in proteinuria is an independent risk factor for ESRD [29]. Proteinuria is strongly associated with arterial stiffness and endothelial dysfunction [30], so proteinuria could reflect arterial stiffness-associated increases. Data from essential hypertension showed arterial stiffness correlated with albumin excretion rate [31]. However, some authors have reported that the 24-h albumin excretion rate did not correlate with AASI [32], and baseline arterial stiffness did not correlate with the incidence of microalbuminuria [33]. A significant relationship between proteinuria and the AASI was not found in our study even we used different statistical methods, which might be related with the complexcity of proteinuria in CKD patients. However, we found AASI correlate with renal function (assessed by eGFR, serum cystanic C and creatinine), which suggested AASI might be a parameter for the assessment for the progression of CKD, and more clinical trials are needed to confirm this finding.

Our study had several strengths. We are the first to report the close relationship between AASI and cardiovascular/renal damage in CKD patients. Also, the sample size was large. Finally, we reported the relationship between AASI and renal cardiovascular damage in Chinese CKD patients. However, we need larger perspective studies to confirm the role of AASI in Chinese CKD patients.

## Conclusions

The present study provided evidence that AASI is associated with renal function and cardiovascular risk factors in Chinese CKD patients. AASI might be a potential index for assessment in CKD patients. More clinical studies with larger cohorts of patients are needed to confirm the role of AASI in Chinese CKD patients.

## Abbreviations

AASI: Ambulatory arterial stiffness index; ARB: Angiotensin receptor blocker; ACEI: Angiotensin-converting enzyme inhibitors; BMI: Body mass index; CCB: Calcium channel blocker; cIMI: Carotid intima-media thickness; eGFR: Estimated glomerular filtration rate; LDL-C: Low-density lipoprotein cholesterol; HDL-C: High-density lipoprotein cholesterol; iPTH: Intact parathyroid hormone; LVEF: Left ventricular ejection fraction; LVMI: Left ventricular mass index; E: Early mitral inflow filling velocity; A: Peak mitral filling velocity at atrial contraction.

## Competing interests

The authors declare that they have no competing interests.

## Authors’ contributions

Conceived and designed the experiments: CW, JZ and TL. Performed the experiments: XL, CL, WG and ZY. Analyzed the data: JZ and CW. Contributed reagents/materials/analysis tools: HP and ZY. Wrote the paper: CW. Review the manuscript for intellectual content: Dr CW, JZ and TL. All authors read and approved the final manuscript.

## Pre-publication history

The pre-publication history for this paper can be accessed here:

http://www.biomedcentral.com/1471-2369/14/257/prepub
